# Substance Use among Adolescents: A Retrospective Study (2017–2018) in the Toxicology Unit, University Hospital of Lille, France

**DOI:** 10.3390/toxics10100594

**Published:** 2022-10-07

**Authors:** Sanaa M. Aly, Ahmed Omran, Jean-Michel Gaulier, Delphine Allorge

**Affiliations:** 1 Forensic Medicine and Clinical Toxicology Department, Faculty of Medicine, Suez Canal University, Ismailia 41522, Egypt; 2 CHU Lille, Unité Fonctionnelle de Toxicologie, F-59000 Lille, France; 3 Pediatrics and Neonatology Department, Faculty of Medicine, Suez Canal University, Ismailia 41522, Egypt; 4 Université de Lille, URL 4483—IMPECS—IMPact de l’Environnement Chimique sur la Santé Humaine, F-59000 Lille, France

**Keywords:** forensic toxicology, alcohol, illicit substances, prescription substances, substance-related death

## Abstract

Research on adolescent substance use is of utmost importance. Using local toxicological data, both prevalence and pattern of substance use (SU) and substance-related death (SRD) can be assessed to design effective prevention programs. A retrospective study of toxicology investigations of all adolescents referred to the medico-legal section of the Toxicology Unit of the University Hospital of Lille, France, for a 2-year period from 2017 to 2018. In the total sample of 1961 cases, adolescents accounted for 3.3% of the cases (n = 65). Among the adolescents, 16.9% were aged 10–14 years and 83.1% were aged >14–19 years. About 69.2% were males. Less than 70% of all presented adolescents used substances. More than two-thirds (74%) of positive detections were male. Illicit substances (43%) were the most detected substance followed by alcohol (20%) and prescription substances (20%). Tetrahydrocannabinol (THC) was extremely common as it was found in 29% of all adolescents. Cocaine and amphetamines were detected in 13.8% of total tested adolescents. Polysubstance use was common between alcohol and THC and among males. About one-third of deaths were due to substance use. About 54% of SRD was associated with polysubstance detection. It is recommended that illicit substances, ethanol, and prescription substances are targeted for testing among adolescents in order to provide appropriate prevention.

## 1. Introduction

Substance use (SU) represents a preventive medicine dilemma in any society. The users may consume different types of substances that have been legal or accepted for public use (alcohol), shared for conditions other than medical use without prescription (prescription substances), or prohibited by law for public use (illicit substance) [1]. SU is considered a risk factor for morbidity and mortality. Substance users suffer a significant risk of premature death in comparison with their contemporaries as previous studies revealed a 6–38 higher mortality rate among substance users [2,3].

According to the definition by the World Health Organization, an adolescent is an individual who is aged between 10–19 years [4]. Adolescence is a tumultuous time full of experimentation and confusion. More often during this period, the acquired habits are accompanied until adulthood, making this time of great importance for preventive intervention [5]. The prevalence of adolescent SU is rampant and creates a conundrum for medical professionals. Initiation of SU at an early age has been associated with long-term physical, behavioral, social, and health risks [6,7]. SU may lead to neurological changes and behavioral consequences that differ from those that happen in adults due to rapid brain development and maturity during this tenuous period [1]. 

Research based on a postmortem database introduces a complementary and different perspective on SU. It includes those persons who do not usually participate in self-reporting studies, and the results depend on actual toxicological analysis; the results of the measurements could temporally connect the toxic substance effect and the fatality [8]. Deceased adolescents offer a picture of the substance-related fatalities which may be helpful to estimate the SU problem in a certain population, identify trends in drug use patterns, and cause of death among the substance users. The number of substance-related deaths (SRDs) could be used to determine SU which is difficult to be estimated in adolescents. Moreover, the toxicological results of living adolescents are another source to estimate the SU among this age group. Specifically, the study aimed: (1) to determine the demographic characteristics of deceased and living adolescents who were investigated in the medico-legal section of the Toxicology Unit of the University Hospital of Lille, France, during 2017–2018, (2) to estimate the prevalence of SU among [deceased/living, male/female, and early/late] adolescents, and (3) to identify the prevalence of SRDs among adolescents.

## 2. Materials and Methods

### 2.1. Sample

**Figure 1:**
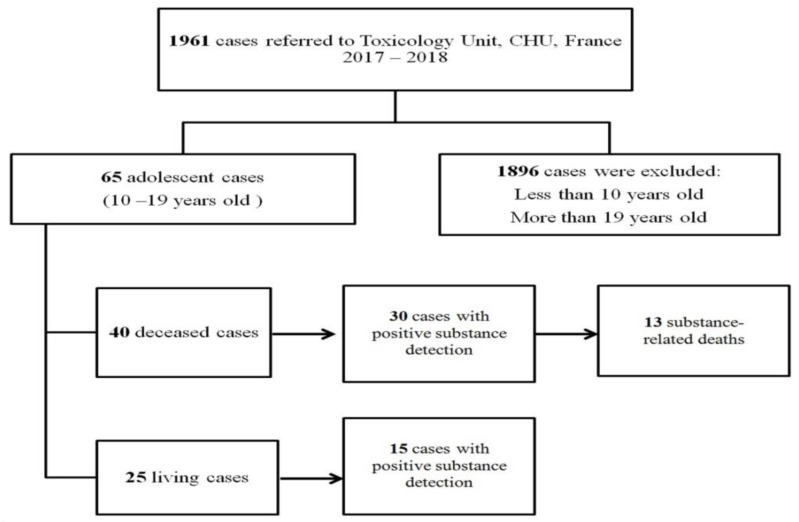
Flow chart showing the selection of adolescent cases referred to the medico-legal section of the Toxicology Unit, University Hospital of Lille (North of France) during 2017–2018.

A systematic retrospective review of adolescent cases (from 10 to 19 years old; deceased and living), who were referred to the medico-legal section of the Toxicological Unit of the University Hospital of Lille (North of France), was carried out over 2 years (2017 to 2018) (Figure 1). Autopsy findings and police department records were used to identify the cause of death, as well as additional relevant information related to drug use, regular treatment, or pathological conditions. The samples were collected either by a forensic pathologist during autopsy in deceased cases or by a medical examiner in living cases. Toxicological analyses of the cases were performed on blood, urine, or other matrices as indicated by case specifics.

### 2.2. Toxicological Analysis

Systematic toxicological investigations that were performed in the collected cases included general unknown screenings using liquid chromatography with the high-resolution mass spectrometry method (LC-HRMS) previously reported [9,10,11]. More selective assays for several classes of therapeutic drugs (paracetamol, psychotropic meds), drugs of abuse (cannabinoids, opiates, amphetamines, cocaine, and new psychoactive substances), or other toxicants (cyanides, alcohols…) were carried out with various *ad-hoc* dedicated methods using liquid chromatography coupled with tandem mass spectrometry detection (LC-MS/MS) or diode array detector (LC-DAD), and headspace-gas chromatography with flame ionization detection (for volatiles including alcohols). The therapeutic, toxic, and fatal concentrations were determined according to the published reference tables [12]. 

### 2.3. Data Recording and Analysis

The cause of death variable has 2 categories, including SRDs and non-substance-related deaths. The age variable was recorded into two categories, 10–14 years of age (defined as early adolescence) and >14–19 years of age (late adolescence). The data analysis is mainly descriptive in nature. Statistics consisted of frequencies and crosstab analysis.

## 3. Results

### 3.1. Sample Demographics

In the total sample of 1961 cases referred to the medico-legal section of the Toxicology Unit of the University Hospital of Lille, France, during 2017–2018, adolescents constituted 3.3% of the cases (n = 65) (Table 1). Among the adolescents, 16.9% (n = 11) were aged 10–14 years and 83.1% (n = 54) were aged >14–19 years. 

Among the adolescents in the present study, 69.2% (n = 45) were males and 30.8% (n = 20) were females. In 2017, 68% (n = 17) were males and 32% (n = 8) were females. In 2018, 70% of the cases (n = 28) were males and 30% (n = 12) were females. 

There were 40 deceased cases whose biological samples were investigated during 2017–2018 (14 in 2017, 26 in 2018). Meanwhile, there were 25 living cases that were investigated during 2017–2018 (11 in 2017, 14 in 2018). In this study, SRDs accounted for 32.5% (n = 13) and 67.5% (n = 27) for non-substance-related deaths. 

### 3.2. Toxicological Analysis

**Figure 2:**
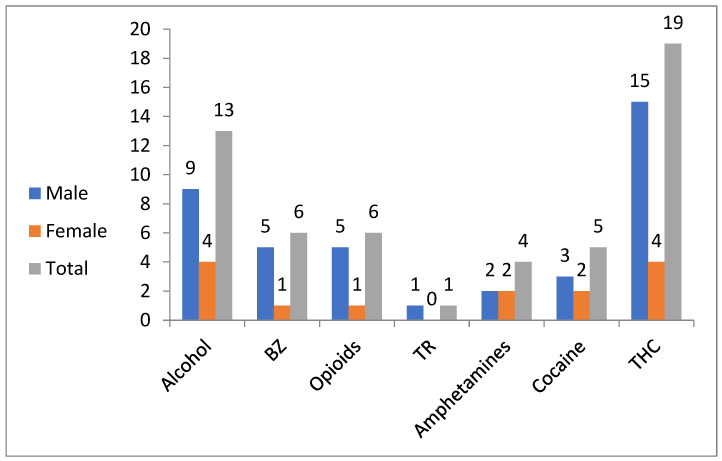
Substance detections among French adolescents (n = 65; 45 males and 20 females) in Lille during 2017–2018. BZ: benzodiazepines; TR: tramadol; THC: tetrahydrocannabinol.

Toxicological data was compiled using all available data from each individual and considered positive if the threshold for positivity was achieved [12]. The most detected substance was THC (Tetrahydrocannabinol) (n = 19, 29.2%), followed by alcohol (n = 13, 20%) and 2 from prescription substances {opioids and benzodiazepine (BZ)} (n = 6, 9%) (Figure 2). 

### 3.3. Age

No substance was detected in the 10–14 years old group (n = 11). This age group consisted of five males and six females. Two out of 11 were living and the other (n = 9) were deceased. The exclusive cause of death among early adolescents was not related to any substance. 

The group aged >14–19 years old (n = 54) consisted of 40 males and 14 females. Twenty-three were living and the other (n = 31) were deceased. The commonest detected substance among late adolescents was THC (n = 19, 35.2%), followed by alcohol (n = 13, 24%). 

### 3.4. Sex

Among males (n = 45), 40 were in the >14–19 years old group. Out of the total number of males in the present study, there were 29 deceased (64.4%). The most common substance detection was THC in 33.3% (n = 15), followed by alcohol in 20% (n = 9) and benzodiazepine-related substances (BZ) in 11% (n = 5) (Figure 2). The SRDs were 31% (n = 9 / 29) of all deaths among males in the present study.

Among females (n = 20), 14 were in the group aged >14–19 years. Out of the total number of females in the present study, there were 11 deceased (55%). The most common substance detection was both THC and alcohol in 20% (n = 4) for each substance followed by both amphetamine and cocaine in 10% (n = 2) for each substance (Figure 2). The SRDs made up 36% (n = 4/11) of all deaths among females in the present study.

### 3.5. Alcohol

In this study, there were 13 cases (13/65 = 20% of all cases) positive for alcohol. All of the cases were in the >14–19 years age group (seven deceased and six living). The majority (9/13 = 69%) were males. The most common cause of death in alcohol-positive cases was substance-related (4/7 57%). Ethanol was quantified in all cases, the cases ranged from 60–2850 mg/L. Ethanol was included in the cause of death for two cases.

### 3.6. Prescription Substances

In the present study, six cases were positive for opioids (6⁄65 = 9% of all cases). The detected substances under this class were morphine, methadone, and codeine. Five cases were males (three died and two living cases) and the last one was a deceased female. All positive opioid cases were in the >14–19 years age group. The sample belonging to living persons (n = 2) were presented to the lab due to the police request after either an accident or violence. The blood analysis of one of them detected morphine (42.3 µg/L), THC (0.8 µg/L), and THC-COOH (20.8 µg/L). For the other living case, blood analysis revealed methadone (2.1 µg/L). The concentration of morphine in the blood of deceased cases in the present study ranged from 5.4 to 73.9 µg/L, with an average of 40.5µg/L (n = 4). The toxicological analysis detected other substances (such as THC, cocaine, and/or BZ) in three positive opioids cases. SRDs were 50% (n = 2/4) among positive opioid adolescents.

Six cases were positive for BZ (6/65, 9%). Four cases were males (three deceased and one living) and two cases were females (one deceased and one living), and all cases were in the >14–19 years age group. Among positive BZ cases (n = 4), the toxicological analysis detected other substances (such as alcohol, THC, cocaine, amphetamine, morphine, and/or codeine). SRDs were 50% (n = 2/4) among deceased positive BZ adolescents.

Tramadol was detected in only one case—a 19-year-old deceased male. The concentrations of TR and their metabolites (*N*-desmethyltramadol, *O*-desmethyltramadol) were 194, 153, and 5.8 µg/L respectively. The cause of death was not substance-related.

Only 61.5% (n = 8/13 of all positive for prescription substance) were positive for one prescription substance, whereas 38.5% (n = 5/13) were positive for poly-prescription use. Of the positive adolescents for prescription substances, 38.5% (5/13) were also positive for illicit substances (THC, cocaine, and their metabolites). 

### 3.7. Illicit Substances

In the present study, 43% (n = 28) were positive for illicit substances (15 out of them were deceased). Only 14% (n = 4/28) of positive illicit substances were positive for poly-illicit (two or three) SU such as THC, MDMA, amphetamine, and/or cocaine. All cases with illicit substance positives occurred in the >14–19 year age group. Males (20/28, 71.4%) were more likely to be positive for illicit substances than females except for MDMA (two females out of three MDMA positive). SRDs constituted 80% (12/15) of deceased cases with positive illicit substances. 

Four cases (6.2%) were positive for amphetamine-related substances. All cases were in the age group of >14 to 19 years old (two deceased and two living cases). The positive amphetamine findings occurred equally in both sexes. Amphetamine (2.8 µg/L) was detected in a 17-year-old living male with polysubstance detection (alcohol, THC, cocaine, and BZ). The blood concentrations of 3,4-Methylenedioxymethamphetamine (MDMA) were 117 and 1890 µg/L and the blood concentrations of MDA were 8 and 31 µg/L. In one of these cases, both MDMA and 3,4-Methylenedioxyamphetamine (MDA) were detected in urine (11600, 760 µg/L). SRDs made up 100% (n = 2/2) of deceased cases with positive MDMA.

Cocaine and/or its metabolites were detected in five cases (7.7%). All cases were in the age group of >14 to 19 years old (four deceased and one living case). The positive cocaine findings occurred in three males and two females. In the present study, the blood concentration of cocaine was 3.4 µg/L (n = 1). For benzoylecgonine (BE), the blood concentrations ranged from 3.4–204 µg/L with a mean = 59.4 µg/L. The blood concentrations of ecgonine methyl ester ranged from 1.3 up to 4.1 µg/L with a mean of 1.8 µg/L. SRDs accounted for 75% (3/4) of deceased cases with positive cocaine substances.

Cannabinoid was the most frequently detected illicit substance (19/65, 29.2% of all cases), which occurred exclusively in the >14–19 years age group (nine deceased and ten living cases). Males comprised 78.9% (15/19) of positive THC cases. The THC blood concentration ranged from 0.7 to 40.5 µg/L, with an average of 6.7 µg/L. The average THC-COOH blood concentration was 10 µg/L, with a range from 0.9 to 47 µg/L. When THC and/or metabolites were detected, the most common cause of death was substance-related (7/9, 77.8%).

### 3.8. Multiple Substances

In this study, there were 16 cases (six living and 10 deceased) positive for multiple substance detection. Out of 16 cases, 12 cases were with polysubstance detection (alcohol, illicit, and/or prescription substances), and the last four cases were with pure poly-illicit substance detections. The most common polysubstance detection was found between alcohol and THC 43.8% (n = 7) of this group. This pattern of intake was presented exclusively among the >14–19 years (n = 16). The males dominated by a margin of 5:2 in comparison with female cases. SRDs constituted 70% (n = 7/10) of deceased adolescents with multiple substance detection.

### 3.9. Substance-Related Deaths

The death due to substance overdose among adolescents is diagnosed as SRD which is different from positive substance detection (Table 1). The SRD was diagnosed in 32.5% (n = 13/40) of all deceased cases and was more common among males (n = 9, 69%) than females (n = 4, 31%). All (n = 13) of these fatalities were in the age group of >14–19 years. About 54% (n = 7/13) had positive polysubstance detection.

## 4. Discussion

SU is a prevalent problem in adolescence [1,13]. The focus on adolescents is of great importance because of their vulnerability [6,14]. The early initiation of SU is highly associated with substance use disorders in adulthood, causing substantial harm to the user and society [6]. Moreover, SU among adolescents has short and long-term repercussions such as poor academic performance, injury, unintended pregnancy, mental disorders, and addiction [15]. The reasons for SU were for getting high, increasing performance, because of peer pressure, or for purposes of self-medication [13]. 

This is a retrospective study of toxicology investigations of (deceased and living) adolescents (10–19 years old) referred to the medico-legal section of the Toxicological Unit of the University Hospital of Lille (North of France) for 2 years from 2017 to 2018. The present study displayed that more than two-thirds (69.2%) of presented adolescents used substances. A previous French study (2006) reported that the prevalence of drug users among a young population of students (n = 3561) from Toulouse University was based on a self-administered questionnaire is 44% [16]. The variance in prevalence could be justified due to the difference in time period and place of study within France. In addition, it has been reported that the postmortem prevalence rates exceed those found with self-reported data. Although the cases in the present study are at high risk of SU due to their forensic referral, it is confirmed that the accuracy of self-reported data is variable and may undermine the magnitude of this epidemic [1].

The positive toxicological results in the present study were found exclusively in a group of late adolescence (>14–19 years old). This is in agreement with the World Drug Report (2018), which revealed that SU is typically initiated in late adolescence and peaks in young adulthood [17]. 

More than two-thirds (74%) of positive detections in the present study were in males. In polysubstance use, males also dominated by a margin of 5:2 in comparison to females. The SRDs occurred more likely among males than females. This is in the same direction as previous studies that confirmed the dominance of males either in SU and its consequences [8].

Illicit substances (43%) were the most commonly detected substance among tested adolescents followed by alcohol (20%) in the present study. THC was extremely common among adolescents as it was found in 29% of all tested cases. Regardless of smoking, a previous French study is in agreement with the present study as it reported that the most consumed products by adolescents are alcohol and cannabis [13]. Another French study revealed the highest prevalence for THC (16.8%) [16]. The main risk of THC is its potential for being a gateway substance to more harmful substances. Moreover, the adolescents who are THC regular users performed poorer on performance tests of learning, cognitive flexibility, visual scanning, error commission, and working memory after 4 weeks of abstinence [1]. This was also in line with a previous Italian study that showed that 74.5% of participating high school students (15–17 years old) were alcohol drinkers [18]. A study from Poland declared that 30.9% of medical students with a mean age of 19 years old were hazardous drinkers [19]. A Spanish study revealed that the prevalence of alcohol and THC use in last month was about 14% and 3%, respectively, among secondary school students [20]. Other illicit substances, such as cocaine and amphetamines, were detected in 13.8% of total tested adolescents in this study. Furthermore, a previous study found minimal use of cocaine (0.6–3.2%) and amphetamines (1%) among French adolescents [13,16]. The difference could be attributed to the change of trend of SU through time and even in different places within the same country.

The prevalence of prescription substances in the present study was 20%. The most common detected prescription substances were opioids (9%) and BZ (9%). According to an earlier French study, 1.9% of people took psychotropic medications, mostly BZ, and <1% used opiates [16]. Another French study considered that opiates and methadone are the most dangerous treatments when misused [13]. It has been reported that the use of mild opioids in France decreased steadily until 2011 (53%) during the 10-year study period (2006–2015). By contrast, the use of strong opioids increased by 37% during the same period [21]. 

The most common polysubstance detection (46.6%) was found between alcohol and THC. Of the positive adolescents for prescription substances, more than three-fourths were also positive for illicit substances or other prescription substances. Polysubstance use is a common pattern among both recreational and regular users. However, polysubstance use among adolescents is linked to an increased risk of developing long-term consequences and engaging in risk-taking through binge drinking or stimulant use such as “ecstasy” [6,22]. In Europe, a wide variation in the patterns of polysubstance use among drug users was reported, ranging from occasional alcohol and cannabis use to the daily use of combinations of heroin, cocaine, alcohol, and BZ [6,17].

In the present study, about one-third of deaths (32.5%) were due to SU. About 54% of SRDs in the current study were associated with polysubstance detection. These results are in agreement with previous reports that revealed the extreme outcome of SU is death. Globally, deaths due to SU increased from 2000 to 2015 by 60%. The top two ranks for recorded deaths among different age groups including adolescents were Europe followed by the Americas [6,17].

## 5. Conclusions

Updated revision regarding commonly used substances and identifying the danger directed at adolescent health is needed to provide appropriate prevention. SU becomes a major problem as the adolescent moves into adulthood. If problems persist, polysubstance use and addiction may occur and increase the complexity of the problem. For this reason, increased supervision and effective prevention programs against SU for adolescents are justified, both for surveillance and reduction of harm.

## Figures and Tables

**Table 1:** Characteristics of adolescents referred to the medico-legal section of the Toxicology Unit, University Hospital of Lille, France (2017–2018).

Variables	Death (n *=* 40)		Alive (n *=* 25)
	Substance-Related (n *=* 13)	Non Substance-Related (n *=* 27)	
**Age group**			
10–14 years old (n = 11)	0	9	2
>14–19 years old (n = 54)	13	18	23
**Sex**			
Male (n = 45)	9	20	16
Female (n = 20)	4	7	9
**Substance detection**			
Positive (n = 45)	13	17	15
Single (n = 29)	6	14	9
Multiple (n = 16)	7	3	6
Negative (n = 20)	-	10	10
**Substance detected**			
Alcohol (n = 13)	4	3	6
Opioids (n = 6)	2	2	2
Benzodiazepines (n = 6)	2	2	2
Tramadol (n = 1)	0	1	0
Amphetamines (n = 4)	2	0	2
Cocaine (n = 5)	3	1	1
Tetrahydrocannabinol (n = 19)	7	2	10

## Data Availability

The anonymized data presented in this study are available on request from the corresponding author.
